# A deep learning approach for medical waste classification

**DOI:** 10.1038/s41598-022-06146-2

**Published:** 2022-02-09

**Authors:** Haiying Zhou, Xiangyu Yu, Ahmad Alhaskawi, Yanzhao Dong, Zewei Wang, Qianjun Jin, Xianliang Hu, Zongyu Liu, Vishnu Goutham Kota, Mohamed Hasan Abdulla Hasan Abdulla, Sohaib Hasan Abdullah Ezzi, Binjie Qi, Juan Li, Bixian Wang, Jianyong Fang, Hui Lu

**Affiliations:** 1 Department of Orthopedics, The First Affiliated Hospital, College of Medicine, Zhejiang University, #79 Qingchun Road, Hangzhou, 310003 Zhejiang Province People’s Republic of China; 2 Department of Rehabilitation Medicine, The First Affiliated Hospital, College of Medicine, Zhejiang University, #79 Qingchun Road, Hangzhou, 310003 Zhejiang Province People’s Republic of China; 3 Department of Infrastructure and General Affairs, The First Affiliated Hospital, College of Medicine, Zhejiang University, #79 Qingchun Road, Hangzhou, 310003 Zhejiang Province People’s Republic of China; 4 UniDT Technology (Shanghai) Co., Ltd, Shanghai, 200436 People’s Republic of China; 5 School of Mathematical Sciences, Zhejiang Univeristy, #38 Zheda Road, Hangzhou, 310027 Zhejiang Province People’s Republic of China; 6 Suzhou Warrior Pioneer Software Co., Ltd. (Room 26, Building 17, No. 6, Trade City, Wuzhong Economic Development Zone), Suzhou, 215000 Jiangsu Province People’s Republic of China; 7 Alibaba-Zhejiang University Joint Research Center of Future Digital Healthcare, Zhejiang University, #866 Yuhangtang Road, Hangzhou, 310058 Zhejiang Province People’s Republic of China; 8 Zhejiang University School of Medicine, #866 Yuhangtang Road, Hangzhou, 310058 Zhejiang Province People’s Republic of China

**Keywords:** Environmental sciences, Health care

## Abstract

As the demand for health grows, the increase in medical waste generation is gradually outstripping the load. In this paper, we propose a deep learning approach for identification and classification of medical waste. Deep learning is currently the most popular technique in image classification, but its need for large amounts of data limits its usage. In this scenario, we propose a deep learning-based classification method, in which ResNeXt is a suitable deep neural network for practical implementation, followed by transfer learning methods to improve classification results. We pay special attention to the problem of medical waste classification, which needs to be solved urgently in the current environmental protection context. We applied the technique to 3480 images and succeeded in correctly identifying 8 kinds of medical waste with an accuracy of 97.2%; the average F1-score of five-fold cross-validation was 97.2%. This study provided a deep learning-based method for automatic detection and classification of 8 kinds of medical waste with high accuracy and average precision. We believe that the power of artificial intelligence could be harnessed in products that would facilitate medical waste classification and could become widely available throughout China.

## Introduction

Medical waste (MW) refers to directly or indirectly infectious, toxic, or otherwise hazardous waste generated by medical institutions during medical or preventative care and related activities, and specifically includes infectious, pathological, damaging, pharmaceutical, and chemical waste^1^. These wastes contain a large amount of bacteria and viruses, and have the potential to cause space pollution, acute viral infection, and latent infection^2^. If they are not properly managed, they can contaminate the surrounding environment, where they pollute the land, water, plants, animals, and air, causing the spread of disease. MW also poses a great threat to the physical and mental health and the quality of life of medical staff and patients^3^.

Currently, MW in China is generally collected and processed centrally by a unified acquisition department, and faces challenges such as inadequate use of waste bins, lack of detailed classification of medical waste or even stacking randomly, and insufficient training of waste classification personnel^4^. High expenses are also one of the reasons for improper disposal of medical waste. Because more processing costs have to be paid to external agencies, the cost of medical waste disposal for hospitals has risen accordingly. The increase in expenditure has caused hospitals to deploy waste disposal facilities and human resources more casually, and in turn, hospitals’ medical waste disposal has steeply declined in quality, which greatly increases the potential for medical waste to contaminate the environment and harm the associated staffs, while reducing the chances of its recycling.

Since the classification of MW varies from country to country^5^, it becomes difficult to count which types of MW are most common. However, in most cases, textile materials, such as gauzes and bandages, occupy the central stage and account for about 83%-97% of overall MW^6,7^. This is followed by plastic products, which account for 39%-49%. Notably, medical plastic waste includes infusion bags, syringe, blood bags, tubing, gloves, labware, and medical packaging-related waste usually made of PVC plastic, and they will produce many toxic substances such as dioxins and furans after incineration, which is by far the most common means of medical waste disposal, thereby, the health of the residents and the atmosphere are at great risk^7^. Sharps such as needles, surgical blades, and broken glass, account for 12% of the total waste generated and are the main cause of injury and infection among medical workers and medical waste handlers^6,8,9^. In this case, the classification of hazardous and common MW is our main concern. Eight types of MW that comprised of textile materials, such as gauze; plastic materials, such as gloves, infusion bags, infusion apparatus, syringes; and sharp objects such as infusion bottles, tweezers, and needles, were selected as the objects of study in an attempt to improve the efficiency and safety of MW classification. Because of their different shapes, it is feasible to classify them by image analysis.

Deep learning-based algorithms have promoted significant advances in waste sorting and medical image analysis ^10,11^. Convolutional neural networks (CNNs) are one of the most important network types in the field of deep learning. The most attractive feature of CNNs is that they can learn increasingly complex features from the input data. For example, the Alex Krizhevsky Network (AlexNet) won the ILSVRC competition in 2012, setting off the current craze of deep learning. Its innovations included the use of ReLU functions, dropout regularization, multi-GPU distributed computing, and data augmentation during training^12^. With the successful application of CNNs in the field of image recognition, Simonyan et al. proposed a simple and effective CNN architecture design principle. Their architecture, called VGG, popularized the idea of using smaller convolutional kernels and deeper network layers (VGG19 has up to 19 layers, AlexNet has up to seven)^13^. GoogLeNet (also known as Inception-V1GoogLeNet) won the 2014 ILSVRC contest, and contained multiple inception modules, each of which applied multiple filters of different sizes to the input; the network then concatenated the results. GoogLeNet also popularized the idea of using global average pooling instead of fully connected layers, thus significantly reducing the number of model parameters and solving the gradient loss problem posed by training deeper networks^14^. In 2015, He et al. proposed ResNet, adding skip connections to the standard path. ResNet retained information as the data passed through the layer. ResNet is 152 layers deep (20 times deeper than AlexNet and 8 times deeper than VGG), and won the 2015-ILSVRC championship^15^. Even with the increase in depth, ResNet's computational complexity is still lower than VGG's. ResNeXt is constructed by repeating a building block that aggregates a set of transformations with the same topology^16^. This simple design results in a homogeneous, multi-branch architecture that has only a few hyper-parameters to set.

**Figure 1:**
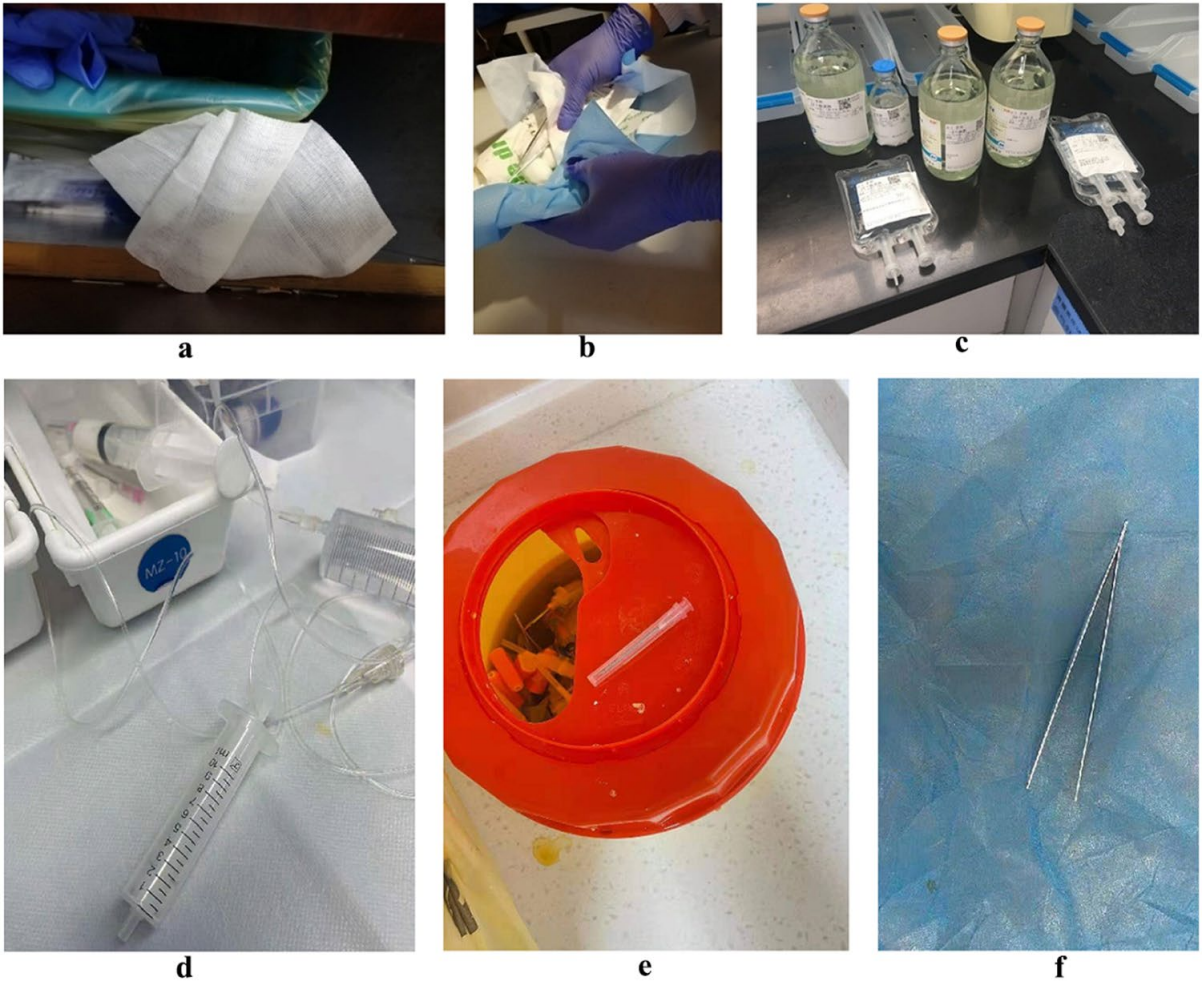
Examples of the medical waste. (**a**) Gauze, (**b**) Gloves, (**c**) Infusion bags and bottles, (**d**) Infusion apparatus and syringe, (**e**) Syringe needles, (**f**) Tweezers.

**Figure 2:**
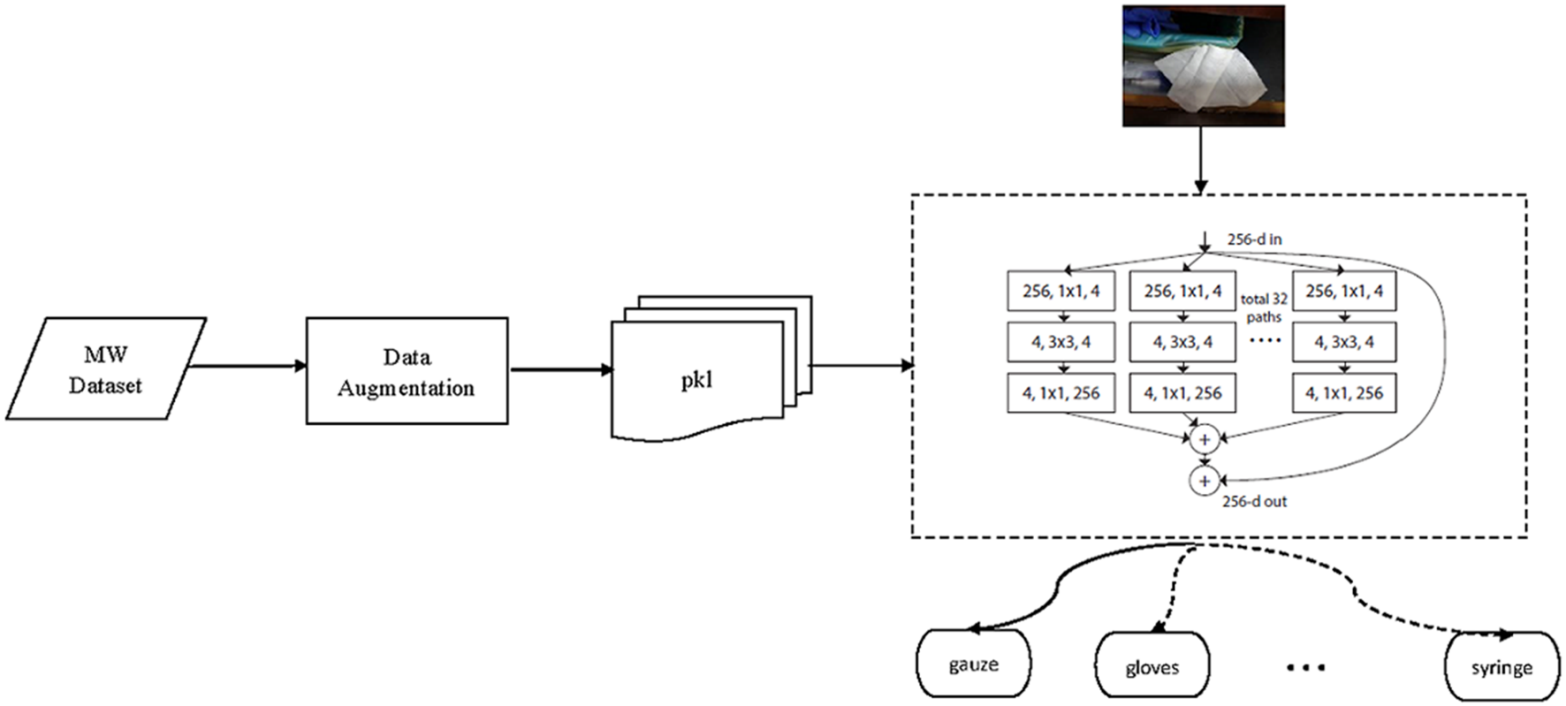
Deep MW: a overview of the deep learning framework.

Machine learning algorithms are widely used in supervised learning, and can solve many practical problems. However, in comparison, the deep neural network (DNN) model has more advantages in image processing and image classification, because deep learning algorithms are better at solving image problems. When people classify medical waste manually, it is done by visually judging the type of waste, which is actually done by image features. Therefore, it makes sense to use the deep learning model to classify medical waste (Figs. 1, 2).

## Results

**Figure 3:**
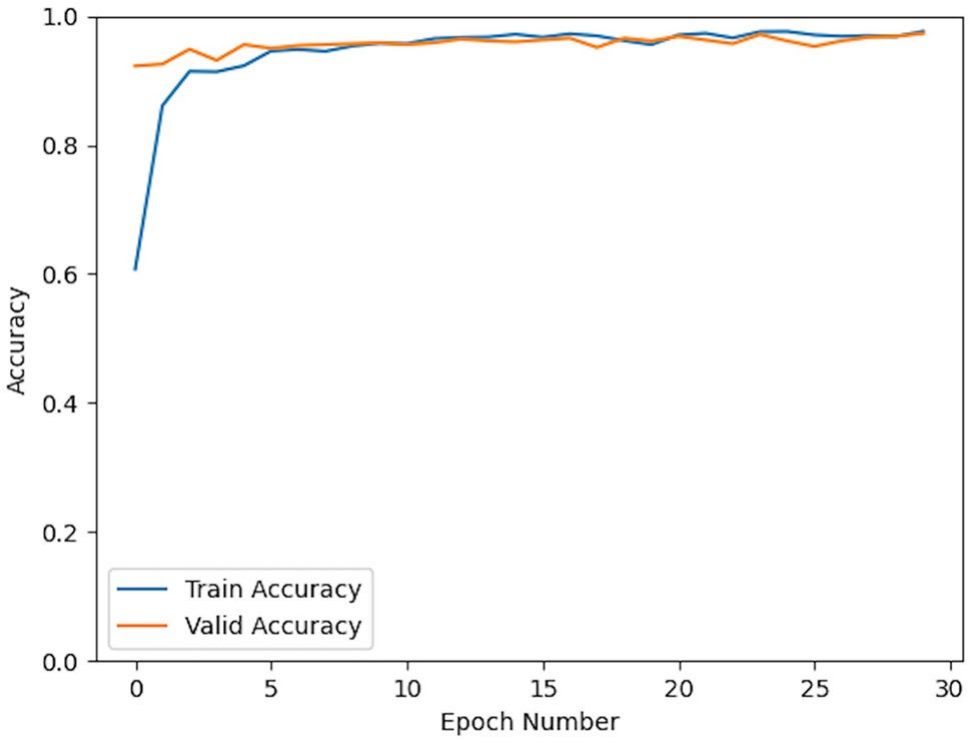
History curves for train accuracy (blue line) and valid accuracy (yellow line).

**Figure 4:**
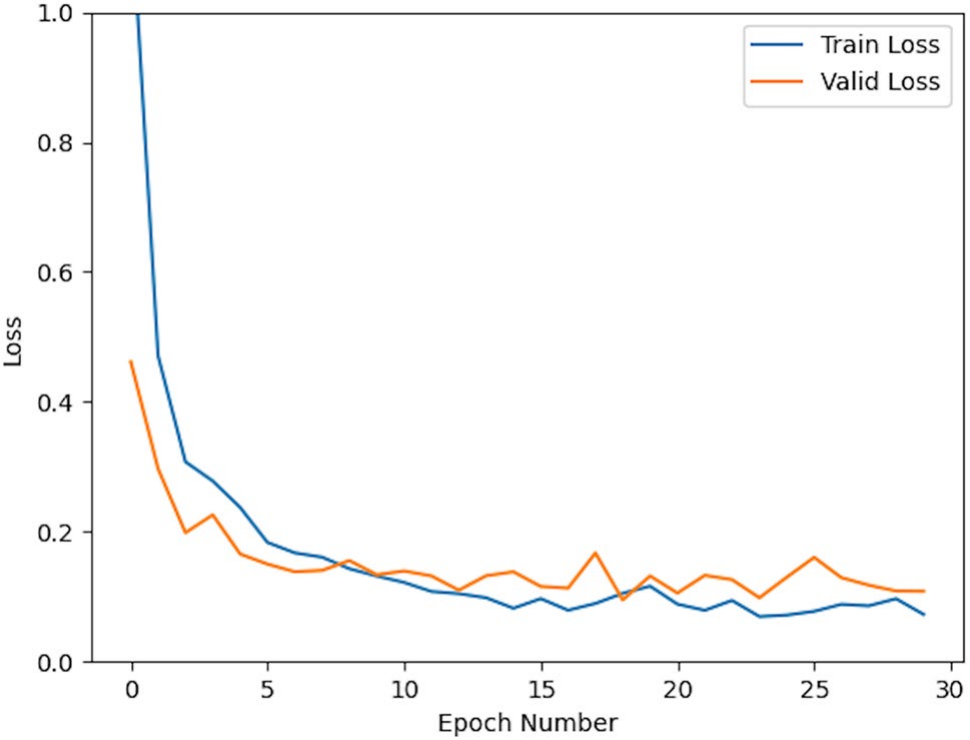
History curves for train loss(blue line) and valid loss(yellow line).

To show the effectiveness of our approach, we conducted a five-fold crossover experiment on the data set. For each experiment we used 2784 images as the training set and 696 as the validation set. The results of the five cross-validation experiments are shown in Table 1. Figures 3 and 4 show the change curves of the loss function and accuracy for each experiment.

**Table 1:** Quantitative results: precision, recall and f1 score with N-fold cross-validation.

Cross-validation experiment	Precision	Recall	F1 Score
First fold	0.97	0.97	0.97
Second fold	0.98	0.98	0.98
Third fold	0.97	0.97	0.97
Forth fold	0.98	0.98	0.98
Fifth fold	0.97	0.97	0.97

**Figure 5:**
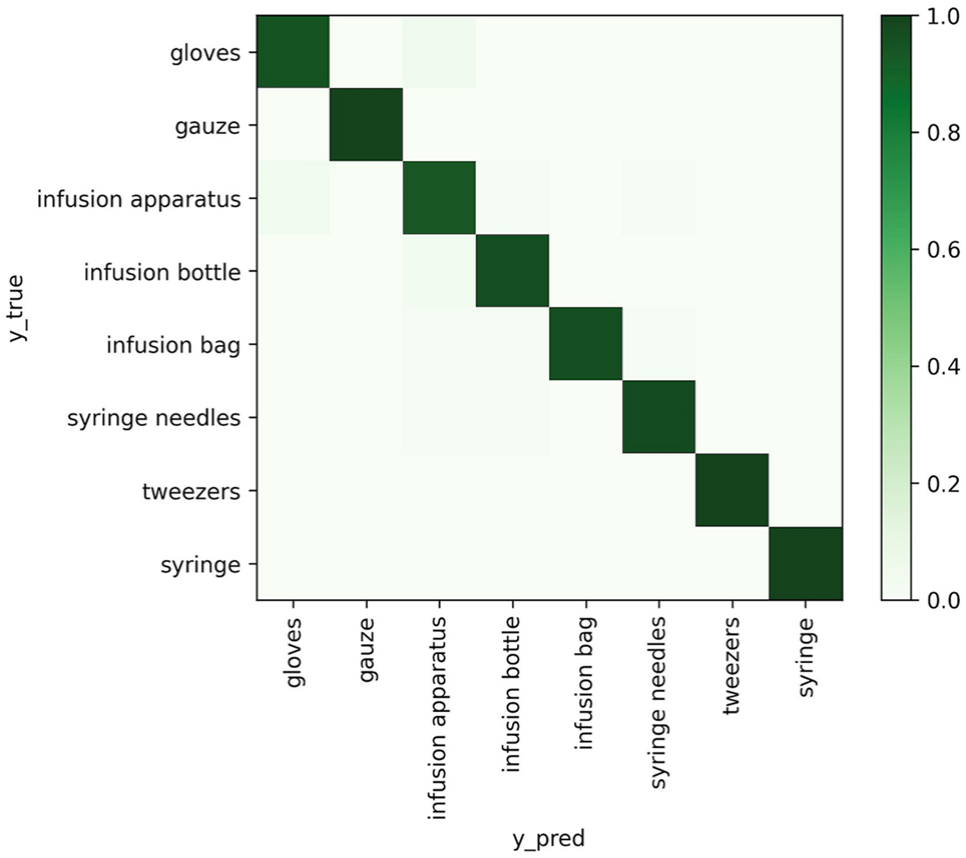
Confusion matrix for the eight categories classification.

For the classification problem, we selected the following indicators to evaluate the model results: f1 score, recall, and precision. These evaluation indicators were calculated by category. In the multi-classification problem, the accuracy of simple calculation was biased, so more detailed evaluation standards were needed to measure the performance of the model. At the same time, we drew a confusion matrix to visualize the classification effect of the five categories [Fig. 5].

Practically, our calculation is performed within the deep learning framework named PyTorch, and for the reason of efficiency, the GPU version with cuDNN computational kernels is applied to accelerate the training procedure. As the traditional classification problem, the cross-entropy loss function is effective in this research, and some extensions on classical ResNeXt-50 are applied for our case: the output size of fully connected layer is fitted to 256, and a dropout layer paramered with 40 percents is applied subsequenty. Finally, the number of final output is set to the same with the categories of the waste. As for the training process, the learing rate is chosen as 1e-3 without decay, and the training process ended within no more than 100 steps. It is also worth to mention that all of the numerical results in this paper is obtained on our GPUs workstation equipped with NVIDIA Tesla V100.

## Discussion

Poor management of medical waste can lead to adverse environmental impacts and human health risk. According to the World Health Organization (WHO), of the total amount of medical waste, 25% of waste is regarded as hazardous and about 75% as non-hazardous. However, both of these types of waste are generally mixed and disposed of together^17^. When hidden in mixed waste, sharp objects such as needles and razor blades can cause injuries to cleaning staff, and waste contaminated with patient bodily fluids not only increases the risk of infection for medical staff, but its improper disposal can also significantly increase the potential for infection in the surrounding population^18,19^. In addition, mixing waste not only increases sanitation labor, but also the burden on environmental protection departments^20^. At present, mixed medical waste is sent to be incinerated, and the significant amount of soot and other emissions it produces pollutes the land and atmosphere ^7,20^.

However, with the progress and development of society, people are more concerned about their health problems, causing the production of medical waste to increase rapidly^21^. In only 3 years, from 2013 to 2016, the annual growth rate of the production of medical waste in China was nearly 20% according to China's Ministry of Environmental Protection. The burden of sorting waste by manpower alone is too great, considering the large number of staff to be hired and the cost of managing and training them. Therefore, we used a CNN to develop Deep MW, an image recognition system for sorting medical waste, which can realize simpler, more efficient and accurate sorting and recycling of medical waste, as well as reduce the risk of occupational exposure for medical waste facility workers [Table S1]. There are other similar contributions, such as iWaste^22^, where for different class of medical waste are detected and classified. In the sense of accuracy of classification, DeepMW has better result and more convenient extending to more categories of objects.

The results presented in the previous section show that advanced AI solutions can be applied for automatic identification and classification of medical waste with a high accuracy, even with a limited imaging dataset. The primary goal of this study was to achieve the highest possible classification accuracy using a data set of only 3480 medical waste images and the developed AI algorithm. The main issue concerned here is to alleviate overfitting dut to the limited training data. To mitigate this problem, various image augmentation techniques are applied, such as rotation, rescaling, clipping and flipping. Noticing that the classical ResNeXt network is a effective and highly modularized architecture for classification tasks, a traditional technique is to repeat the building block with the same topology to formation a set of transformations, as well as used in our previous work^23^. This simple design results in a homogeneous, multi-branch architecture that requires only a few hyper-parameters. In a practical calculation, it is also effective to increase the cardinality instead of using deeper and wider structure. The current task is one of the fundamental tasks in the whole practical processing. In a practical using, It is always suggested to capture/detect the object from the video frames, which depend on a very efficient detection network to perform online detection. The classification task is the subsequently job after the image is captured and cropped from the video frames. In this stage, the accuracy is the concerned issue, while the speed is the main issue at the stage of detection. In this sense, we formulate the problem as a classification task.

The mixed packing of medical waste treats garbage as waste, whereas the separate packaging of garbage treats waste as a resource. For instance, plastics or polystyrene in medical waste can be widely recycled to produce secondary products such as accessories, packaging, cases, and containers. Recycling and reuse can save a remarkable amount of energy when compared with the virgin material^24^. For example, the air pollution and water pollution produced when waste paper is restored and recycled are much less than the pollution produced when the original natural fiber is used to make paper. The classification of medical waste can also bring certain economic benefits to the hospital. On the one hand, medical products currently in use usually have high-quality paper packaging, so paper recycling is very feasible. On the other hand, disposal of medical waste is very expensive. After sorting, this portion of the expenditure can be reduced and indirect economic benefits can be generated. Another reason to focus on medical waste identification is that it promotes safe disposal by taking simple steps to pack and separate garbage, as recyclable waste is distributed to various recycling departments, and does not pollute the soil and atmosphere. Classification of medical waste also helps improve the environmental protection awareness of staff. With the current goals of advocating environmental protection and creating a conservation-oriented society, if every employee in the operating room cares about environmental protection, saves resources, and contributes to environmental protection, they can establish a tradition of conservation and enhance the public image of the hospital. Therefore, medical waste classification can not only reduce medical harm, but also promote recycling, reduce consumption, and bring economic benefits to medical and health services providers.

In summary, we propose a deep learning-based method, Deep MW, for automatic detection and classification of medical waste based on images. Our results show that the method is capable of identifying the 8 main kinds of medical waste with high accuracy and average precision. The application of the method described in this paper to medical waste could prevent hazardous and DEA-regulated medications from being commingled with non-hazardous medications. This in turn would reduce the volume of medical waste generated by medical programs and the associated transportation and disposal costs.

## Materials

### Description of the dataset

For this research, we used a medical waste dataset from The First Affiliated Hospital, Zhejiang University, collected in 2019. This special dataset consists of labels, image data, and medical waste boarder for 3480 samples, which could be categories into eight kinds of medical waste. In this sense, the proposed deep learning approach is named with Deep MW (deep medical waste).

There was some abnormal data in the dataset, as shown in Fig. 1. In addition, some images contained more than one category of waste. For example, the upper left corner of the gauze in Fig. 1a contains gloves, an infusion bottle and infusion bag are both in Fig. 1c, and infusion apparatus and syringe both appear in Fig. 1d. This type of image had a lower degree of accuracy than classical images. The quality of the source image, such as size and background, could influence the performance of classification model siginificantly. In practical application, additional operation on the source image is nessary, such as local sampling^25^, cropping^26^, etc. We used data preprocessing to ensure sufficient data quality and consistent sample size.

Following the suggestions from medical experts, eight categories of medical waste are considered as the typical illustration in this research respectively were gauze, gloves, infusion bags, infusion bottles, infusion apparatus, syringe needles, tweezers and syringe. More specifically, the dataset classification and corresponding sample size are shown in Table 2.

**Table 2:** Dataset category and corresponding sample size.

Classification	Sample size
Gauze	508
Gloves	440
Infusion bag	443
Infusion bottle	433
Infusion apparatus	426
Syringe needles	410
Ttweezers	451
Syringe	369
Total dataset size	3480

It is not difficult to see from the table that the sample size of the eight kinds of medical waste was basically balanced: sample gauze had the most images (508), and sample syringe the fewest (369). The sample size distribution was workable for algorithm classification.

### Data augmentation

We augmented the training set by generating new medical waste images in order to balance the classes, and adjusted them using zoom, rotation, shear, translation, flipping, Gaussian noise, and stretching transformations. We applied these transformations to each image, with parameters chosen at random. We then included the new images in the training set, while images of the most favored class were excluded at random until the classes were balanced.

### Dividing the dataset

First, we randomly (without repeated sampling) divided the dataset into ten parts, using nine of them in turn as training sets and one as the test set. Next, we augmented the training set as described above and conducted experiments. Finally, we averaged the results of ten experiments as an estimate of the overall accuracy of the algorithm.

## Methods

### Samples

We collected images of 8 kinds of typical medical waste: gauze, gloves, infusion bags, infusion bottles, infusion apparatus, syringe needles, tweezers, and syringe. Since the images for the sampling are collected by ourselves, the category balance is taken into account at this stage, in which the samples’ sizes are kept almost in the same level. The number of images of each type was: 508 of gauze, 440 of gloves, 443 of infusion bags, 433 of infusion bottles, 426 of infusion apparatus, 410 of syringe needles, 451 of tweezers, and 369 of syringe sets. The dataset includes all the image forms of each category collected so far, if there are samples of non-single medical waste images, adding them will help to improve the accuracy of the algorithm, can be considered enough in scope. The total number of training sets was 3480. For practical usage, the enrichment of the categories of the classification is simple to modify with the current framework. More categories could be appended by preparing more images for the interested objects.

### Framework of the data flow

We packaged the training sets and tested them to generate PKL files. Then, we inputted the PKL files to the pre-training model, and the loss function was used to iteratively train the network to obtain the automatic classification model. After the model training was completed, the medical waste images were either predicted by the prediction script, or subjected to batch classification prediction using the verification script. For a better illustration, let us depicted the framework of proposed deep learning system, namely DeepMW, within the following Fig. 2.

### Setup of the deep neural network

ResNeXt follows the principle of concise design^16^. Multiple modules with the same structure are repeatedly divided, and each module aggregates the grouping transformation of the same topology. This simple design produces a homogeneous, multi-branch architecture, which requires only a few hyperparameters.

Each module of ResNeXt follows two simple rules: (i) if generating feature maps of the same size, the modules share the same hyperparameters (ii) each time the feature map is downsampled by 2 times, the width of the module is multiplied by 2 times. The second rule ensures that the calculation complexity of FLOP (floating-point operations, multiply–add operations) is approximately the same for all blocks. Using these two rules, we only need to design a template module to determine all modules in the network accordingly. Therefore, these two rules greatly reduce design complexity, allowing us to focus on some key factors.

A neuron is the basic unit of an artificial neural network. It mainly performs inner product operations, that is, nonlinear transformation completed by a fully connected layer and a convolutional layer. In simple terms, the inner product operation can be viewed as a combination of the three operations of splitting, transforming, and aggregating. (i) splitting: decompose the input vector x into low-dimensional embeddings. Generally, each low-dimensional embedding corresponds to a one-dimensional subspace x_i_. (ii) transforming: transform the low-dimensional embedding obtained by decomposition, that is, multiply the weight ${w}_{i}$ to get ${w}_{i}{x}_{i}$

(iii) aggregating: the transformations in all embeddings are aggregated by ${\sum }_{i=1}^{D}$

Formally, we present aggregated transformations as

$$F\left(\mathrm{x}\right)= \sum_{i=1}^{C}{T}_{i}(x)$$

where ${T}_{i}(x)$ can be an arbitrary function. In ResNeXt, it is best to use a network instead of the basic transformation (${w}_{i}{x}_{i}$). Broadly speaking, transformation is not limited only to a function; it can also be a network, that is, a “Network-in-Neuron” structure. Similar to a simple neuron, ${T}_{i}$ should project $x$ into an (optionally low-dimensional) embedding and then transform it.* C* is the size of the transformation set to be aggregated, and we refer to *C* as cardinality. All ${T}_{i}$ have the same topology. We set each transformation ${T}_{i}$ as a bottleneck structure. Thus, the first 1*1 layer of each ${T}_{i}$ produces low-dimensional embedding.

We converted the aggregate transformation into a residual function

$$y=x+ \sum_{i=1}^{C}{T}_{i}(x)$$

where y is the output.

The result of determining whether an item was medical waste was outputted in principal binary. The cross-entropy loss function for input was reasonable from the perspective of classification. Our label for medical waste showed whether the item was medical waste or not. The expression of the loss function was as follows.

$$L\left(x\right)= \sum_{i=1}^{batch\_size}\mathrm{log}({p}_{l\left(x\right)}\left(x\right))$$

where ${w}_{l(x)}$ corresponds to the weight of different labels and l(x) is the label type of pixel point x.

### Transfer learning

Image or medical examination classification is one of the first fields where deep learning has made a significant contribution. In medical examination classification, one or more images are usually used as input, and a single diagnostic result is used as output (for example, whether there is a disease). In this scenario, each diagnosed case is a sample. Compared with data sets in the field of computer vision, data sets for medical examinations usually have a small sample size (hundreds vs. thousands and millions of samples). Transfer learning is required to meet the needs of deep learning for large data sets.

In practical applications, the following two transfer learning strategies are often used: (1) Using a pre-trained network without modification as a feature extractor. (2) Fine-tuning a network trained with medical data. The advantage of the former strategy is that there is no need to train a deep network and the extracted features are directly input into the existing image analysis process. Both strategies are popular and have been widely used. However, few authors have conducted the thorough investigations necessary to arrive at the best strategy. For more details of these strategyies, we are referred to a recenty survey^27^. These two studies pre-trained Google’s Inception v3 network on medical data and achieved performance comparable to human experts.

As far as the authors are aware, using only a pre-trained network as a feature extractor has not yet achieved similar results. In this study, we also used a pre-training strategy to implement our algorithm. Let us show some numerical results in the following Table 3 on the performance with or without fine-tune.

**Table 3:** Comparisons on Accuracy, Recall and F1-score between the pertained model and the fine-tune model.

	Precision		Recall		F1-score	
	Pretrain	Fine-tune	Pretrain	Fine-tune	Pretrain	Fine-tune
Gloves	0.90	0.93	0.94	0.88	0.92	0.90
Gauze	0.95	0.98	1.00	1.00	0.98	0.99
Apparatus	0.94	0.93	0.84	0.94	0.89	0.94
Bottle	0.84	0.98	0.75	1.00	0.79	0.99
Bag	0.79	1.00	0.98	0.98	0.88	0.99
Needles	0.91	1.00	0.83	0.98	0.87	0.99
Tweezers	1.00	0.98	0.90	1.00	0.95	0.99
Syringe	0.91	0.98	0.98	1.00	0.94	0.99
Averaged	0.91	**0.97**	0.90	**0.97**	0.90	**0.97**

Significant values are in bold.

In the above experinments, the pretrained model is supplied by the deep learning framework directly, and it is pretrained in practice with the ImageNet dataset. While the Fine-tune model is archived by performing further training, through which the model parameters are adjusted accordingly for better accuracy on our destinating images. It is not difficult to find out from the numerical results that, the fine-tune model performs better on each category in consideration.

Actually, some kinds of medical wastes, such as tweezers and infusion bottle, are similar in some sense with certain common objects, and it does have some benefits to perform fine-tuning from such pretrained models. On the other hand, the accuracy of pretrained model is not satisfied on some soft object, such as gloves and bags. It may caused by the possibility of their folding shape.

## Supplementary Information

Supplementary Information.

## Data Availability

The dataset supporting the conclusions of this article is included with the article.

## Abbreviations

MW: Medical waste
CNN: Convolutional neural network
AlexNet: Alex Krizhevsky network
Deep MW: Deep medical waste
FLOP: Floating point operations, multiply and add operations
