# Supplementary material for: A deep learning approach for medical waste classification
Source: Sci Rep. 2022 Feb 9;12:2159. doi: 10.1038/s41598-022-06146-2 (PMC8828884; doi:10.1038/s41598-022-06146-2)
Supplement: Supplementary file 1 — Supplementary Information. [file 41598_2022_6146_MOESM1_ESM.docx]

Table S1: [Convolutional neural network based garbage classification systems]

| **Article** | **Type of algorithm** | **Type of waste** | **Classification** | **Quantity** | **Accuracy** |
| --- | --- | --- | --- | --- | --- |
| *Deep MW(our system)* | *ResNet* | Medical waste | *Gauze, gloves, infusion bags, infusion bottles, infusion apparatus, syringe, syringe needles, tweezers* | *3480 pictures* | *97.2%* |
| Chen J ^1^ | R3D+C2D network | Medical waste | Gloves, hairnet, mask, and shoecover | 970 videos. Each video is 5 second long and has a frame rate of 24 FPS. | 79.99% |
| Togacar M ^2^ | Combination of Convolutional Neural Network and AutoEncoder | Solid waste | Organic and recyclable wastes | 25,077 pictures | 99.95％ |
| Mao WL ^3^ | DenseNet121 | Solid waste | Cardboard, glass, metal, paper, plastic, and trash | 2527 pictures | 99.6％ |
| Funch OI ^4^ | Convolutional Neural Network | Consumer trash bags | Glass and metal | 2000 | 98% |
| Sreelakshmi K ^5^ | Capsule-Net | Solid waste | Plastic and non-plastic | Dataset 1: 12893 statistics collected from public places | 96.3% |
|  |  |  |  | Dataset 2: 6134 statistics collected from private environment. | 95.7% |
| Huang GL ^6^ | Combination classification model based on VGG19, DenseNet169, and NASNetLarge | Municipal Solid Waste | Paper, glass, metal, plastic, textile, and organic waste and 12 subcategories | Dataset 1: collected manually from Google search, | 96.5% |
|  |  |  |  | Dataset 2: selected from the existing published image databases. | 94% |
| Meng S ^7^ | DenseNet | Solid waste | Glass, paper, cardboard, plastic, metal, other trashes | 2527 pictures | 94.1% |
| Davis P ^8^ | VGG-16 | Construction waste material | Second fix timbers, shuttering/ formwork timbers, shuttering/ formwork ply and particleboards, hard plastics, soft plastic, bricks and concrete, cardboards and polystyrene | 1758 pictures | 94％ |
| Fallati L ^9^ | commercial software – PlasticFinder | Anthropogenic Marine Debris on the beach | Lighter, bottle, straw, net, plastic bag, aluminum can, platic containers,plastic utensils, flip flop, ohter | 697 picturess | 94% |
| Cao L ^10^ | InceptionV3 | Domestic waste | Recyclable waste, hazardous waste, household food waste and residual waste | 6000 pictures | 93.2% |
| Zheng H ^11^ | Combination classification model based on GoogLeNet, ResNet-50, and MobileNetV2 | Household solid waste | Trashnet dataset: cardboard; glass; metal; paper; plastic; trash | 2527 pictures | 93.50% |
|  |  |  | FourTrash dataset: wet waste, recyclables, harmful waste, and dry waste | 47332 pictures | 92.85% |
| Chu Y ^12^ | Multilayer hybrid deep-learning system | Personal waste in public areas | Recyclable or the others | 5000 pictures | 91.6% |
| Nowakowski P ^13^ | R-CNN | Waste electrical and electronic equipment | Refrigerators, washing machines, and monitors or TV sets | 210 picturess | 90％-97％ |
| Bobulski J ^14^ | AlexNet network(23 layer); | Plastic waste | Polyethylene terephthalate, high-density polyethylene, polypropylene and polystyrene | 33 000 simulated images per class | 93.45-99.23% |
|  | CNN author proposed(15 layer) |  |  |  | 80.23-91.72% |
| Ruiz V ^15^ | VGG-16 | Domestic garbage | Glass, paper, cardboard, plastic, metal, and general trash | 2,527 picturess | 76.94% |
|  | Resnet |  |  |  | 79.32% |
|  | VGG-19 |  |  |  | 87.71% |
|  | Inception |  |  |  | 88.66% |
|  | Inception-Resnet |  |  |  | 88.34% |
| Adedeji O ^16^ | ResNet-50 | Solid waste in the urban area | Glass, metal, paper, plastic | 1989 pictures | 87% |
| Wang H ^17^ | VGG-19 | Bulky wastes | 95 types | 69,737 pictures | 86.19％ |
| Kylili K ^18^ | VGG-16 | Plastic marine litter | Bottles, buckets, and straws | 750 picturess | 86% |
| Kumar S^19^ | YOLOv3 | Solid waste in the urban area | Cardboard, glass, metal, paper, plastic and organic waste | 7826 pictures | 85.29％ |
| Cai Y ^20^ | VGG-16 | Cotton trash | Block fiber included plastic film and feather, dead cotton, hang, leaf, linear fiber contained polypropylene and hemp rope, stalk | 7,786 augmentation sample | 84.14％ |
| Sakr GE ^21^ | AlexNet | Solid waste | Plastic, paper and metal | 2000 pictures | 83％ |
| Rajak AAR ^22^ | AlexNet | Solid waste in the urban area | Metal, paper, plastic and non-recyclable waste | Unknown | 80% |
| Altikat A ^23^ | Deep convolutional neural networks with four layers | Solid waste | Paper, glass, plastic, and organic waste | 450 pictures | 61.67% |
|  | Deep convolutional neural networks with five layers |  |  |  | 70% |
| Watanabe JI ^24^ | YOLO v3 | Marine debris | Underwater sea life and debris floating on the ocean | Unknown | 69.6% |
| Rad MS ^25^ | OverFeat-GoogLeNet model | Wastes on the Streets | 1. Beverage and meal packages, 2. Cigarettes and derivatives, 3. Leaves, 4. Newspapers and papers, 5. Vegetable waste, etc. | 469 pictures | 63.2% |
| Politikos DV ^26^ | R-CNN | Seafloor marine litter | Plastic bags, plastic bottles, plastic sheets, plastic cups, cans, fishing nets, plastic small plastic sheets, tires, big objects, plastic caps, unspecified | 1166 picturess | 62% |

All data in the table are from the original article

## References

1 Chen, J., Mao, J., Thiel, C., Wang, Y. & Ieee. in *42nd Annual International Conferences of the Ieee Engineering in Medicine and Biology Society: Enabling Innovative Technologies for Global Healthcare Embc'20* *IEEE Engineering in Medicine and Biology Society Conference Proceedings* 5794-5797 (2020).

2 Togacar, M., Ergen, B. & Comert, Z. Waste classification using AutoEncoder network with integrated feature selection method in convolutional neural network models. *Measurement* **153**, doi:10.1016/j.measurement.2019.107459 (2020).

3 Mao, W.-L., Chen, W.-C., Wang, C.-T. & Lin, Y.-H. Recycling waste classification using optimized convolutional neural network. *Resources Conservation and Recycling* **164**, doi:10.1016/j.resconrec.2020.105132 (2021).

4 Funch, O. I., Marhaug, R., Kohtala, S. & Steinert, M. Detecting glass and metal in consumer trash bags during waste collection using convolutional neural networks. *Waste Management* **119**, 30-38, doi:10.1016/j.wasman.2020.09.032 (2021).

5 Sreelakshmi, K., Akarsh, S., Vinayakumar, R., Soman, K. P. & Ieee. in *2019 5th International Conference on Advanced Computing & Communication Systems* *International Conference on Advanced Computing and Communication Systems* 631-636 (2019).

6 Huang, G.-L., He, J., Xu, Z. & Huang, G. A combination model based on transfer learning for waste classification. *Concurrency and Computation-Practice & Experience* **32**, doi:10.1002/cpe.5751 (2020).

7 Meng, S., Zhang, N., Ren, Y. & Publishing, I. O. P. in *5th Annual International Conference on Information System and Artificial Intelligence* Vol. 1575 *Journal of Physics Conference Series* (2020).

8 Davis, P., Aziz, F., Newaz, M. T., Sher, W. & Simon, L. The classification of construction waste material using a deep convolutional neural network. *Automation in Construction* **122**, doi:10.1016/j.autcon.2020.103481 (2021).

9 Fallati, L. *et al.* Anthropogenic Marine Debris assessment with Unmanned Aerial Vehicle imagery and deep learning: A case study along the beaches of the Republic of Maldives. *Science of the Total Environment* **693**, doi:10.1016/j.scitotenv.2019.133581 (2019).

10 Cao, L. & Xiang, W. *Application of Convolutional Neural Network Based on Transfer Learning for Garbage Classification*. (2020).

11 Zheng, H. & Gu, Y. EnCNN-UPMWS: Waste Classification by a CNN Ensemble Using the UPM Weighting Strategy. *Electronics* **10**, doi:10.3390/electronics10040427 (2021).

12 Chu, Y. *et al.* Multilayer Hybrid Deep-Learning Method for Waste Classification and Recycling. *Computational Intelligence and Neuroscience* **2018**, doi:10.1155/2018/5060857 (2018).

13 Nowakowski, P. & Pamula, T. Application of deep learning object classifier to improve e-waste collection planning. *Waste Management* **109**, 1-9, doi:10.1016/j.wasman.2020.04.041 (2020).

14 Bobulski, J. & Kubanek, M. in *Advances in Computational Intelligence, Iwann 2019, Pt Ii* Vol. 11507 *Lecture Notes in Computer Science* (eds I. Rojas, G. Joya, & A. Catala) 350-361 (2019).

15 Ruiz, V., Sanchez, A., Velez, J. F. & Raducanu, B. in *From Bioinspired Systems and Biomedical Applications to Machine Learning, Pt Ii* Vol. 11487 *Lecture Notes in Computer Science* (eds J. M. F. Vicente *et al.*) 422-431 (2019).

16 Adedeji, O. & Wang, Z. in *2nd International Conference on Sustainable Materials Processing and Manufacturing* Vol. 35 *Procedia Manufacturing* (eds T. C. Jen, E. Akinlabi, P. Olubambi, & C. Augbavboa) 607-612 (2019).

17 Wang, H. *et al.* Smartphone-based bulky waste classification using convolutional neural networks. *Multimedia Tools and Applications* **79**, 29411-29431, doi:10.1007/s11042-020-09571-5 (2020).

18 Kylili, K., Kyriakides, I., Artusi, A. & Hadjistassou, C. Identifying floating plastic marine debris using a deep learning approach. *Environmental Science and Pollution Research* **26**, 17091-17099, doi:10.1007/s11356-019-05148-4 (2019).

19 Kumar, S. *et al.* A Novel YOLOv3 Algorithm-Based Deep Learning Approach for Waste Segregation: Towards Smart Waste Management. *Electronics* **10**, doi:10.3390/electronics10010014 (2021).

20 Cai, Y., Wu, J., Zhang, C. & Ieee. in *Proceedings of the 38th Chinese Control Conference* *Chinese Control Conference* (eds M. Fu & J. Sun) 8783-8788 (2019).

21 Sakr, G. E. *et al.* *Comparing Deep Learning And Support Vector Machines for Autonomous Waste Sorting*. (2016).

22 Rajak, A. A. R., Hasan, S. & Mahmood, B. Automatic waste detection by deep learning and disposal system design. *Journal of Environmental Engineering and Science* **15**, 38-44, doi:10.1680/jenes.19.00023 (2020).

23 Altikat, A., Gulbe, A. & Altikat, S. Intelligent solid waste classification using deep convolutional neural networks. *International Journal of Environmental Science and Technology*, doi:10.1007/s13762-021-03179-4 (2021).

24 Watanabe, J.-i., Shao, Y. & Miura, N. Underwater and airborne monitoring of marine ecosystems and debris. *Journal of Applied Remote Sensing* **13**, doi:10.1117/1.Jrs.13.044509 (2019).

25 Rad, M. S. *et al.* in *Computer Vision Systems, Icvs 2017* Vol. 10528 *Lecture Notes in Computer Science* (eds M. Liu, H. Chen, & M. Vincze) 195-204 (2017).

26 Politikos, D. V., Fakiris, E., Davvetas, A., Klampanos, I. A. & Papatheodorou, G. Automatic detection of seafloor marine litter using towed camera images and deep learning. *Marine Pollution Bulletin* **164**, doi:10.1016/j.marpolbul.2021.111974 (2021).
